# *Kochujang*, fermented soybean-based red pepper paste, decreases visceral fat and improves blood lipid profiles in overweight adults

**DOI:** 10.1186/1743-7075-10-24

**Published:** 2013-02-26

**Authors:** Youn-Soo Cha, Soo-Ran Kim, Ji-Ae Yang, Hyang-Im Back, Min-Gul Kim, Su-Jin Jung, Won O Song, Soo-Wan Chae

**Affiliations:** 1 Department of Food Science and Human Nutrition, Chonbuk National University, Obesity Research Center, 664-14 Dukjin-Dong 1-Ga, Jeonju, Jeonbuk, 561-756, Republic of Korea; 2 Clinical Trial Center for Functional Foods, Chonbuk National University Hospital, 634-18, Geumam-dong, Deokjin-gu, Jeonju, Jeonbuk, 561-172, Republic of Korea; 3 Department of Pharmacology, Chonbuk National University, Medical School, Jeonju, Jeonbuk, 561-172, Republic of Korea; 4 Department of Food Science and Human Nutrition, Michigan State University, East Lansing, MI, 48824-1224, USA

**Keywords:** *Kochujang* (KCJ), Visceral fat, Triglyceride, Atherosclerosis index, Apolipoprotein

## Abstract

**Abstract:**

Health benefits of *Kochujang* (KCJ) and its bioactive compounds have been reported in several in vitro and animal studies.

**Objective:**

The aim of this study was to investigate the efficacy of KCJ supplementation on body composition and blood lipid profiles in overweight adults.

**Methods:**

Sixty overweight men and women with BMI ≥23 kg/m^2^ and waist-hip-ratio (WHR) ≥0.90 for men and ≥0.85 for women were randomly assigned to a KCJ supplement (n=30, 32 g/day) or placebo (n=30, 32 g/day) group for a 12-week, double-blind, placebo controlled study. We measured anthropometric parameters, serum lipid profiles, abdominal fat distribution by computerized tomography and calculated the atherosclerosis indices in 53 subjects (n=26 in *KCJ* group, n=27 in placebo group) who completed the study.

**Results:**

After 12 weeks, the KCJ group showed a significant reduction in visceral fat (cm^2^) (*p*<0.05), although body weight (kg) and WHR did not change. Serum concentration of triglycerides and ApoB were decreased when compared to those of the placebo group.

**Conclusion:**

KCJ supplementation (32 g/day) for 12 weeks in overweight adults showed anti-atherosclerotic and anti-obesogenic effects.

**Trial registration:**

Clinical trials.gov Identifier: NCT01532375

## Background

The increasing westernized Korean dietary lifestyle, including frequently eating away from home, has favored foods that have not been a part of the traditional Korean diet [1]. The changing consumers’ demand for western food products and diminished traditional dietary lifestyle have overlapped with the prevalence of obesity and obesity-related chronic diseases in Korea [2,3]. *Kochujang* (KCJ), a fermented soybean-based red pepper paste, has long been one of the most representative and commonly used seasonings in Korean cuisine as a sauce, dressing or seasoning for meat, vegetable dishes, stew and soup. The KCJ is produced by fermenting powder red peppers combined with powdered meju (fermented soybean powder), salt, malt-digested rice syrup, and rice flour for about six months. The fermentation process extends the storage period while increasing bioavailability of bioactive ingredients [4] such as free amino acids, peptides, alcohols, organic acids, capsaicin and flavonoids [5,6]. KCJ has unique flavors of sweet and hot red pepper combined with savory soybean protein hydrolyzate and nucleic acids. In recent years, KCJ has gained its popularity outside Korea for its taste and health benefits derived from the several ingredients [7-16] that are produced by the fermentation process [17-22]. The functional substances either singularly or in combinations have exhibited anti-obesogenic, anti-oxidative, and anti-mutagenic properties in several *in vitro* experiments and in various murine models [7-16]. Anti-obesogenic and anti-atherogenic properties of fermented soy products have been demonstrated in obese adults [23], possibly through modulation of hepatic acyl-CoA synthase, carnitine palmitoyltransferase I, and acyl-CoA oxidase [24]. Recently Ludy and Mattes [25] reported that hedonically acceptable doses of red pepper altered thermogenesis and appetite. Their findings are consistent with previous reports of Reinbach et al [26] and others [27] who reported the alteration of appetite and energy balance as a result of red pepper or capsaicin intake. Lee et al [28] suggested that the alterations could be through changes in orexigenic and anorexigenic neuropeptides in hypothalamus.

With the epidemic of obesity and diabetes growing around the world, KCJ could be potentially effective in preventing and treating obesity and cardiovascular risks [16-20] if proven in humans. To date however no clinical trials of human feeding studies have been reported with KCJ supplementation. In the present randomized, double-blind, placebo-controlled clinical trial, we tested the hypothesis that KCJ supplementation decreases body fat and improves blood lipid profiles in overweight adults.

## Subjects and methods

### Study subjects

Healthy men and women volunteers, 19 to 65 years of age, with BMI ≥23 kg/m^2^ and WHR of > 0.90 for men and >0.85 for women participated in the study. Excluded from the study were individuals with (1) lipid metabolic disorders; (2) >10% changes in body weight in the past 3 months; (3) cardiovascular disease such as arrhythmia, heart failure, myocardial infarction, and wearing pacemaker; (4) allergy or hypersensitivity to any of the ingredients in the test products; (5) history of reaction to any of the experimental products or of gastrointestinal diseases such as Crohn’s disease or gastrointestinal surgery (caecum or enterocele surgery); (6) participation in other clinical trials within the past 2 months; (7) abnormal hepatic liver function, renal disease such as acute/chronic renal failure, nephrotic syndrome; (8) use of anti-psychosis drug therapy within 2 months; (9) laboratory test, medical or psychological conditions deemed by the investigators to interfere with successful participation in the study; (10) history of alcohol or substance abuse; and (11) pregnancy or breastfeeding. All subjects willfully signed consent to participate in the study after receiving a detailed explanation of the purpose with research procedures. The research protocol was approved by the Institutional Review Board of Chonbuk National University Hospital's *Clinical Trial Center for Functional Food*.

### Study design

At the onset of the study, each subject was interviewed for demographic information such as sex, date of birth, age and other lifestyle factors such as past smoking, drinking and medical histories. The subjects were divided into a KCJ group (n=30) and a placebo group (n=30) for the 12-week randomized, double-blind, placebo-controlled design. The KCJ supplement (32 g/day in pills) was equivalent to the usual daily intake of 39g wet weight of KCJ as consumed by Koreans. The KCJ used in the study was produced by the standardized manufacturing process and ingredients. Then a single batch KCJ was lyophilized and made into pills for the entire study (Imshil Herbal Medicine Co, Imsil, Republic of Korea). The placebo supplement had the same appearance and caloric contents without the principal ingredients that are present in KCJ (Table 1).

**Table 1:** **Composition of *Kochujang *and placebo supplements (g/day)**

	**Kochujang**	**Placebo**
Glutinous rice flour (g)	10.9	-
Powdered red pepper(g)	11.9	-
Malt (g)	5.2	-
Powdered fermented soybeans (g)	4.7	1.1
Spicy flavor powder(g)	-	0.1
Perfume of red pepper(g)	-	0.3
Honey(g)	-	1.4
Caramel pigment(g)	-	0.1
Salt (g)	5.2	1.4
Soy sauce (g)	2.1	6.7
Powdered cowpeas (g)	7.5	9.3
Powdered cocoa (g)	2.5	1.4
Powdered vegetable fat(g)	-	6.3
Wheat flour(g)	-	13.1
Red colors(g)	-	1.5
Total wet weight (g)	50	43
Freeze-dried weight (g)	32	32
Energy (kcal)	114	113.1

The subjects were instructed to maintain their usual lifestyle and activity levels and avoid other functional foods or dietary supplements during the 12-week study period. The subjects visited the clinic every 4 weeks for a total of five clinic visits (initial screening, and at weeks 0, 4, 8, 12) for monitoring and assessment of compliance with the protocol. At both the beginning and at the end of the 12-week intervention; anthropometric and biochemical parameters, computed tomography, vital signs, and dietary intakes were measured for both KCJ and placebo groups. At each visit to the clinic, the subjects were asked about adverse effects experienced, changes in physical activity, lifestyle, eating patterns, and pill compliance.

### Anthropometric measurements

Height was measured using a DS-102 (JENIX, Korea). Weight, BMI, % body fat, body fat mass, muscle mass, and WHR were measured using bioelectrical impedance analysis (Inbody 3.0, Biospace Co., Seoul, Korea). At weeks 0 and 12, visceral fat, subcutaneous fat, and total fat were also measured using computed tomography (CT) scans and visceral to subcutaneous ratios (VSR) were calculated. Having lumbar vertebrae 4 (L4) as the center, five different regions between -20 and +20 were photographed for the abdominal fat area calculation. Visceral fat and subcutaneous fat were divided after setting the boundary between the abdominis and the peritoneum. After computing the numeric value of the total fat and visceral fat area, the subcutaneous fat area was calculated by subtracting the area of visceral fat from the total fat. The proportion was calculated as VSR.

### Serum lipid, lipoprotein, atherosclerosis and index in glycaemic control factor

Fasted blood samples (>12 hr) were used to assess the blood lipid profiles: total cholesterol (TC), triglycerides (TG), high-density lipoprotein cholesterol (HDL), low-density lipoprotein cholesterol (LDL), free fatty acid (FFA), apolipoprotein AI (ApoA_1_), apolipoprotein B (ApoB), fasting plasma glucose, and HbA1c. Blood tests were conducted with a Hitachi 7600-110 analyzer (Hitachi High-Technologies Corp., Tokyo, Japan) by standard methods [23] used in the clinical laboratory of *Chonbuk National University Hospital*. Atherosclerosis Indices (AI) were measured by calculating the ratios (TC-HDL)/HDL, LDL/HDL and ApoB/ApoA1. The Cardiac Index (CI) was also calculated by the TC/HDL ratio.

### Safety and dietary assessment

**Safety measurements** for the subjects were taken by electrocardiogram, hematology test and blood chemistry tests, i.e., white blood and red blood cell counts, hemoglobin, hematocrit, platelet count, total protein, albumin, alanine aminotransferase (ALT), aspartate aminotransferase (AST), blood urea nitrogen (BUN), and creatinine levels [29]. Pulse and blood pressure were measured at each visit after a 10-minute rest, using the OMRONT4 digital blood pressure monitor (OMRON Corp., Tokyo, Japan). Each subject completed a 3-day dietary record for two weekdays and one weekend day in order to evaluate the energy intake and diet quality at each clinic visit. The twenty-four hour dietary intake data were analyzed by one dietitian throughout the study using Can-Pro 3.0 software (The Korean Nutrition Society, Seoul, Republic of Korea).

### Statistical analysis

Statistical analyses were performed using SAS version 9.0 for Windows (SAS Institute, Cary, NC, USA) and SPSS for Windows, version 16.0 (SPSS, Chicago, USA). Data were expressed as the mean and standard errors (SE).

The statical analysis for the main analysis were performed according to the intention-to –treat principle. Sample size for the study was based on the mean (SE) cm^2^ of visceral fat difference between treatments in the previous study [30], -7.8 (3.6) cm^2^ for the experimental group and +3.9 (6.4) cm^2^ for the placebo group. It was estimated to provide 80% power to detect a difference between groups in visceral fat of 11.7(SD; 20.5) cm^2^ with α = .05, using a 2-tailed *t*-test of the difference between means. The minimum sample size was determined to 48 participants (24 per group) by calculated, to allow for a 20% dropout rate a total of 60 participants were selected.

Between subjects t-tests were calculated for all variables measures to determine whether there were changes associated with the treatment group. Within each treatment group, paired comparison t tests were calculated to test whether the change from 0-week to 12-week. Repeated measures mixed model analysis of variance was performed to see whether there were effects associated with time (with-person variable), treatment group (between–group variable), or the interaction of time and treatment group.

## Results

### Study subjects

**Figure 1:**
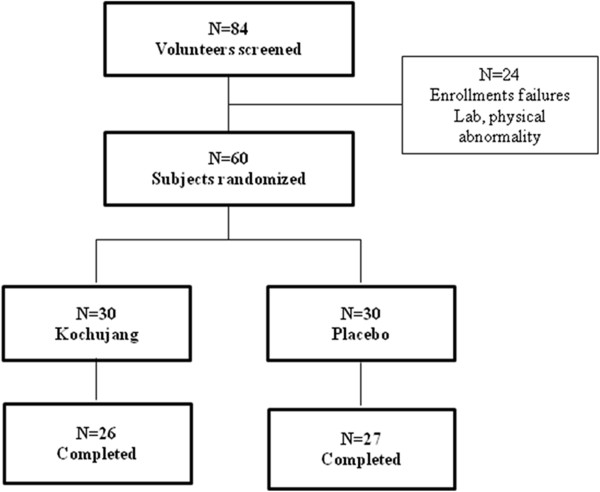
The Flow chart of the study.

The sampling and trial profiles are summarized in Figure 1 along with the number of subjects who completed the study. Out of 84 subjects pre-screened by interviews at the onset, 24 subjects did not meet the selection criteria either in laboratory tests and/or physical examinations. The remaining 60 subjects were randomly assigned to the KCJ (*n=*30) and placebo (*n=*30) groups. Four subjects (13%) from the KCJ group and three subjects (10%) from the placebo group failed to complete the study. Five subjects were disqualified because of inadequate intake of the prescribed supplements, and two subjects voluntarily withdrew due to personal reasons. A total of 53 subjects (KCJ=26, placebo=27) completed this study.

### General characteristics of subjects and dietary assessment

The KCJ and placebo groups were similar in baseline characteristics (Table 2). Significant differences between the two groups were observed in dietary intakes (calorie, carbohydrate, total protein) (Table 3). But no significant interactions (treatment* time) were observed in the dietary intake of macro-and micro-nutrients.

**Table 2:** Baseline characteristics of the subjects

**Variables**	**Kochujang group (n=30)**	**Placebo group (n=30)**	**P-value**^**a**^
Age (y)	42.1±1.3^1^	43.0±2.2	0.786
Sex (M/F) ^**b**^	4/26	3/27	0.688^b^
Weight (kg)	68.0±1.5	570.2±2.3	0.431
BMI (kg/m^2^)	26.7±0.4	27.4±0.8	0.383
WHR	0.91±0.005	0.90±0.006	0.242
Visceral fat (cm^2^)	79.0±5.1	78.3±4.4	0.926
Subcutaneous fat (cm^2^)	250.5±23.6	249.6±16.0	0.977
SBP (mmHg)	118.1±2.43	119.4±2.2	0.681
DBP(mmHg)	74.4±1.7	75.7±1.5	0.563
Pulse rate(bpm)	68.7±1.9	71.49±1.8	0.216
FPG(mg/dl)	83.3±1.6	81.6±1.5	0.449
HbA1c(%)	5.4±0.1	5.4±0.1	0.894
TC (mg/dL)	192.4±4.7	195.3±6.0	0.649
TG (mg/dL)	118.4±10.2	122.0±12.1	0.703
HDL (mg/dL)	52.0±1.7	52.5±2.1	0.822
LDL (mg/dL)	123.6±4.4	125.12±5.8	0.835
FFA (μEq/L)	612.4±40.5	549.5±33.9	0.239
ApoA1 (g/dL)	1.44±0.03	1.45±0.04	0.770
ApoB (g/dL)	0.86±0.03	0.87±0.04	0.944

^1^ Values are expressed as means ± SE.

^a^ By independent t test.

*BMI* body mass index, *WHR* waist to hip ratio, *TC* total cholesterol, *TG* triglyceride, *HDL* high density lipoprotein cholesterol, *LDL* low density lipoprotein cholesterol, *FFA* free fatty acid, *ApoA1* Apolipoprotein A1, *ApoB* Apolipoprotein B., *SBP* systolic blood pressure, *DBP* diastolic blood pressure, *FPG* fasting plasma glucose.

^b^ Chi-squate test. Statistical analyses were run with or without the men in the study, and also controlled for the gender as a covariate.

**Table 3:** **Nutrient intakes of the *Kochujang *and placebo groups at the 0-wk and at 12-wk of the study**

**Nutrients**	**Kochujang group (n=30)**			**Placebo group (n=30)**						
	**0wk**	**12wk**	Change^*^	**0wk**	**12wk**	Change^*^	**P value**^**1**^	**P value**^**2**^	**P value**^**3**^	**P value**^**4**^
Calorie(kcal/d)	1418.5±53.44	1393.1±92.9	-25.4±115.5	1527.1±58.3	1548.1±79.8	20.9±89.9	0.754	0172	**0.028**	0.680
Carbohydrate(g/d)	222.8±8.2	208.2±10.3	-14.6±14.3	242.8±11.0	233.3±12.8	-9.5±16.5	0.816	0.070	**0.030**	0.840
Total protein(g/d)	55.2±2.8	51.2±3.6	-3.9±4.9	60.7±2.2	57.9±5.0	-2.7±5.6	0.867	0.853	**0.040**	0.823
Total lipid(g/d)	34.7±3.8	34.6±3.8	-0.1±4.7	37.6±1.7	37.3±3.5	-0.3±3.4	0.973	0.955	0.927	0.273
Fiber(g/d)	17.8±0.9	14.6±0.8	-3.2±1.3	19.3±1.1	18.3±1.4	-0.9±1.5	0.261	0.823	**0.032**	0.273
Cholesterol(mg/d)	210.5±22.8	249.4±27.5	39.0±27.7	204.3±14.5	280.0±26.4	75.7±31.8	0.385	0.940	0.448	0.429
Na(mg/d)	3,630.4±247.4	3,079.9±225.3	-550.4±305.5	3,665.9±191.2	3697.1±220.4	31.1±280.3	0.168	0.316	0.164	0.170

All values are expressed as means ± SE.

P value^1^, *t*-test between changes of the Kocujinag and placebo group : P value^2,3,4^ time, group and interaction(time*group) effect of two-way repeated measure ANOVA, after adjusting for age and gender.

^*^ Change = 12 wk – 0wk.

† Paired *t*-test between 0-wk and 12-wk *(P<0.05)*.

### Body weight and components

Body weight and body composition data of both KCJ and placebo groups are summarized in the upper part of Table 4. After 12 weeks of KCJ supplementation, no significant changes were observed in body weight, BMI, body fat mass (kg), body fat (%) and WHR.

**Table 4:** **Change of body weight,body composition,abdominal fat area, *index in glycaemic control factor *and blood pressure measurements at the 0-wk and 12-wk of the study**

**Parameters**	**Kochujang group (n=30)**			**Placebo group (n=30)**						
	**0wk**	**12wk**	Change^*^	**0wk**	**12wk**	Change^*^	**P value**^**1**^	**P value**^**2**^	**P value**^**3**^	**P value**^**4**^
Body weight (kg)	66.6±1.4	66.6±1.4	0.06±0.3	68.5±2.4	68.8±2.5	0.3±0.3	0.577	0.140	0.244	0.439
Body fat mass (kg)	22.3±0.7	22.0±0.8	-0.3±0.3	24.5±1.6	24.5±1.6	0.0±0.0	0.476	0.480	0.172	0.391
Body fat (%)	33.4±0.8	33.0±0.9	-0.4±0.4	34.7±.0.9	34.6±0.9	-0.1±0.2	0.498	0.351	0.391	0.413
BMI	26.5±0.4	26.4±0.4	-0.1±0.1	27.4±0.8	27.5±0.9	0.03±0.0	0.606	0.692	0.248	0.544
WHR	0.91±0.006	0.89±0.009	-0.1±0.1	0.90±0.007	0.90±0.01	0.1±0.009	0.351	0.692	0.475	0.274
Total fat (cm^2^)	333.4±29.6	280.6±28.8	-52.8±33.3	324.8±17.9	**272.1±12.6**^**†**^	-52.8±6.2	0.998	0.187	0.680	0.972
Visceral fat (cm^2^)	81.1±5.5	**76.2±5.6**^**†**^	-4.8±3.3	78.4±4.8	78.9±4.3	0.4±2.1	**0.043**	**0.001**	0.974	**0.038**
Subcutaneous fat (cm^2^)	252.3±27.1	204.4±26.7	-47.9±33.4	246.4±17.0	**193.2±11.1**^**†**^	-53.2±6.6	0.875	0.107	0.649	0.869
VSR	0.37±0.03	**0.42±0.03**^**†**^	0.05±0.02	0.36±0.01	**0.44±0.03**^**†**^	0.08±0.01	0.218	**0.001**	0.849	0.302
SBP (mmHg)	117.3±2.4	120.3±2.4	2.9±2.4	119.3±2.3	119.7±2.4	0.4±1.5	0.367	0.260	0.706	0.447
DBP(mmHg)	74.5±1.9	75.4±1.9	0.9±1.9	75.6±1.6	76.9±2.1	1.3±1.5	0.880	0.877	0.548	0.813
Pulse rate(bpm)	68.8±2.1	68.4±1.6	-0.4±1.3	71.4±1.8	71.5±2.0	0.1±1.4	0.748	0.506	0.272	0.726
FPG(mg/dl)	84.6±1.6	80.7±1.8	-3.8±1.1	81.6±1.6	79.6±1.9	-1.9±1.0	0.217	0.056	0.454	0.188
HbA1c(%)	5.42±0.03	5.42±0.08	0.007±0.05	5.42±0.01	5.47±0.01	0.05±0.06	0.607	0.567	0.673	0.681

All values are expressed as means ± SE.

*WHR* waist to hip ratio, *VSR* Visceral adipose tissue to subcutaneous adipose tissue ratio.

P value^1^, *t*-test between changes of the Kocujinag and placebo group : P value^2,3,4^ time, group and interaction(time*group) effect of two-way repeated measure ANOVA, after adjusting for age and gender.

^*^ Change = 12 wk – 0wk.

† Paired *t*-test between 0-wk and 12-wk *(P<0.05)*.

### Abdominal fat distribution by computerized tomography

Abdominal fat distributions measured by computerized tomography are shown in the lower part of Table 4. After 12-week of intervention, the KCJ group showed a significant reduction in visceral fat area compared to the placebo group. Furthermore, significant differences were observed in the visceral fat area from baseline to 12 weeks in both groups. The KCJ group attained a difference of -4.8cm^2^ whereas placebo group showed 0.4 cm^2^ changes in the visceral fat area. Both the change in visceral/subcutaneous fat ratio decreased from baseline to 12 weeks (p<0.05). Furthermore, a significant interaction (time * group) were observed in the visceral fat area (p<0.038).

### Blood lipid profiles and atherosclerosis index

As shown in Table 5, the mean difference in TG, ApoA1 and ApoB from baseline to 12 weeks were -17.9, -0.18 and -0.17, respectively in the KCJ group compared with 13.5, -0.14 and -0.12, respectively in the placebo group. A significant difference in the TG (*p*=0.026) level was observed between the two groups. Along with a significant interaction (time *group) in the difference of TG (*p*=0.042) between the two groups.

**Table 5:** **Serum lipid profiles of the *Kochujang *and placebo groups at the 0-wk and at 12-wk of the study**

**Parameters**	**Kochujang group (n=30)**			**Placebo group (n=30)**						
	**0wk**	**12wk**	Change^*^	**0wk**	**12wk**	Change^*^	**P value**^**1**^	**P value**^**2**^	**P value**^**3**^	**P value**^**41**^
Total cholesterol(mg/dL)	193.4±5.1	189.8±5.9	-3.6±3.9	196.4±6.3	197.9±5.1	1.5±4.2	0.378	**0.047**	0.740	0.367
**Triglycerides(mg/dL)**	112.5±9.1	**94.6±5.8**^**†**^	-17.9±8.5	125.6±12.7	139.1±13.8	13.5±12.9	**0.049**	0.412	**0.026**	**0.042**
HDL (mg/dL)	52.3±1.9	51.2±1.9	-1.07±1.5	52.3±2.2	50.9±1.8	-1.32±1.7	0.912	0.100	0.923	0.670
LDL (mg/dL))	124.9±4.7	122.3±5.2	-0.25±0.14	125.9±6.1	123.6±5.3	-0.15±0.14	0.630	0.188	0.874	0.960
Free fatty acid (μEq/L)	631.8±43.2	604.7±37.4	-27.1±53.9	542.2±34.7	631.9±43.0	89.8±44.0	0.098	0.658	0.669	0.170
ApoA1 (g/dL)	1.44±0.03	**1.26±0.03**^**†**^	-0.18±0.03	1.45±0.04	**1.31±0.03**^**†**^	-0.14±0.03	0.284	**0.002**	0.729	0.216
ApoB(g/dL)	0.87±0.03	**0.70±0.03**^**†**^	-0.17±0.02	0.88±0.04	**0.76±0.04**^**†**^	-0.12±0.02	**0.049**	**0.001**	0.540	**0.025**
Atherosclerosis Indices										
TC/HDL	3.8±0.1	3.4±0.2	-0.42±0.2	3.9±0.2	3.8±0.2	-0.10±0.2	0.303	0.935	0.460	0.357
LDL/HDL	2.4±0.1	2.2±0.1	-0.25±0.1	2.5±0.2	2.3±0.2	-0.15±0.1	0.630	0.976	0.671	0.752
(TC-HDL)/HDL	2.8±0.1	2.5±0.2	-0.32±0.2	2.9±0.2	2.8±0.2	-0.04±0.03	0.253	0.975	0.454	0.297
ApoB/ApoA1	0.61±0.02	**0.56±0.03**^**†**^	-0.05±0.01	0.61±0.03	0.60±0.03	-0.01±0.02	0.296	0.075	0.240	0.593

All values are expressed as means ± SE.

*HDL* High-density lipoprotein cholesterol, *LDL* Low-density lipoprotein cholesterol, *ApoA1* Apolipoprotein A1, *ApoB* Apolipoprotein B, *TC/HDL* total cholesterol/high density lipoprotein cholesterol ratio, *LDL/HDL* low density lipoprotein/cholesterol high density lipoprotein cholesterol ratio, *(TC-HDL)/HDL* (total cholesterol - high density lipoprotein cholesterol)/high density lipoprotein cholesterol ratio;

*ApoB/ApoA1* Apolipoprotein B/Apolipoprotein A1.

P value^1^, *t*-test between changes of the Kocujinag and placebo group : P value^2,3,4^ time, group and interaction (time*group) effect of two-way repeated measure ANOVA, after adjusting for age and gender.

^*^ Change = 12 –wk – 0-wk.

† Paired *t*-test between 0-wk and 12-wk *(P<0.05)*.

Apo B level showed no changes in the baseline and 12weeks in either group, however a significant difference was observed (p=0.049) between the two groups. While no significant interaction (time*group) were found in Apo B levels. There was no significant changes or differences observed in serum TC, HDL, LDL, ApoA1, ApoB and FFA level between the two groups. The changes in TC/HDL and (TC-HDL) /HDL ratio (Table 5) from baseline to 12-week were 0.42 and -0.32, in the KCJ group compared with -0.10 and -0.04, in the placebo group, however, these changes were not significant. ApoB/ApoA_1_ ratio also showed no changes between the baseline and 12weeks in either group, but a reduction in the ratio was observed in the KCJ groups from baseline to 12 weeks.

### Safety measurements

The overall safety measures obtained before and after the intervention indicated no significant changes during the study (data not shown). The evaluations were also expanded to laboratory tests, electrocardiogram and vital signs (blood pressure, pulse) during the subjects’ visits.

## Discussion

We hypothesized that KCJ supplementation for 12 weeks would reduce body fat and improve blood lipid profiles in overweight adults, as demonstrated in animal studies [20-22]. In this clinical trial, we found no significant effects on body weight (kg), body fat (%) or WHR after 12 weeks of KCJ supplementation. However, CT scans did reveal significant reductions in visceral fat area after KCJ supplementation. Furthermore, visceral fat area of the KCJ group was significantly lower than that of the placebo (Table 4). In our study we did not control the lifestyle of the subjects. Apart from the KCJ group, the placebo group also showed changes in certain parameter like total fat and subcutaneous fat, however these changes were not significant. Placebo causes psychological effects rather than the effect caused by its ingredients [30].

Many studies evaluate the effects of specific bioactive compounds or ingredients such as isoflavones, capsaicine, capsioids and red pepper power in subjects as adjuncts with additive effects of dietary intervention in a group of subjects following a weight loss program. This study took a different approach. The present study aimed to examine the biological outcome in humans resulting from the synergistic effects of functional compounds that constitute KCJ as a whole food, rather than those of the individual components in KCJ. We therefore evaluated the effect of supplementing a traditional food, KCJ, along with the diet of overweight subjects following a normal diet and lifestyle. Our subjects did not experience a great degree of overall weight loss as seen in some studies using higher doses of bioactive compounds, but did experience a very significant decrease in visceral fat with concomitant improvements in risk factors for cardiovascular disease (Table 4). Visceral adiposity is known to be associated with an elevated ApoB/ApoA_1_ ratio with is considered to be a risk factor for metabolic syndrome [31]. Therefore, our results showing decreased visceral adiposity and decreased ApoB is consistent with a protective effect of KCJ against the development of metabolic syndrome. Our findings are similar to those in other studies in which adults (30~60 y of age) who ingested capsinoid (6 mg/d) orally lost abdominal fat and had increased fat oxidation [32]. In a study of obese college women who consumed red pepper extracts, the subjects lost both body weight and body fat [33]. The college women were closely monitored for their dietary intake through repeated education and weekly reminders. The close monitoring of dietary intake is believed to have resulted in changes in body weight and body fat composition.

Other studies have also reported that daily supplementation of L-carnitine (300 mg) and isoflavones (400 mg) for 12 weeks decreased visceral fat [34]. Wu et al (2006) reported that isoflavon supplementation for one year resulted in no differences in body mass or body fat, but trunk fat mass decreased significantly [35]. Apart from single bioactive components supplement mediated anti-obesity effect, whole food like fermented soybean paste *doenjang* also reported to have anti-obesity effect in human obese subjects [36]. A Study using fermented kimchi powder as whole food mixed in animal diet proved to be beneficial in limiting weight gain in rodent models [37].

Although statistically insignificant, the changes in mean energy intake in the KCJ group decreased over 12 weeks while that in the placebo group increased (Table 3). It is thus possible that capsaicin or other bioactive components in KJC might have functioned as appetite suppressants. Serum TG levels decreased in the KCJ supplemented group in contrast to those of the placebo group (Table 5). Similar results have been reported when overweight women were supplemented with L-carnitine and isoflavon. Capsaicin supplementation (0.014% wt or 3mg %) with a high fat diet in mice has also been reported to decrease blood TG, with no changes in TC levels [38]. In this study, KCJ supplementation resulted in beneficial effects on TG and ApoB(p<0.05). A study on healthy adults with Kimchi supplementation reported no changes in the LDL-C/HDL-C [39], but supplementation of isoflavon for 12 weeks significantly decreased the (TC-HDL)/HDL [34]. The profound effect of KCJ on visceral adiposity and atherogenic indices observed in the present study might have been mediated through one or more of the bioactive compounds in KCJ such as capsaicin in red pepper, isoflavon aglycones, and peptides from fermented soy [40]. Capsaicin has been reported to upregulate lipolysis and thermogenesis while suppressing appetite [25-27]. Isoflavones such as genestein increases lipid oxidation with upregulated CPT-1 and activation of PPAR-α [21,41,42].

As the people with obesity and overweight tend to develop hypertension, the salt content of KCJ (Table 1) may be of concern. According to KNHANES study [43] (2010), the daily salt intake of all Koreans (including children and the elderly) averages 12.1g/day (4,878 mg Na). In the present study salt intake of the adult subjects from regular meals (~ 8.5 g/day) and KCJ supplement (~ 6 g/day) was ~14.5 g/day (5,800 mg Na) which is 16.55% higher than the mean daily intake by all Koreans. Sodium excretion of the two groups in the present study, before and after the intervention, did not show any significant change.

KCJ could be helpful in utilizing it as ketchup or sauce in western diets. It can be used an a spicy ingredient in many dishes worldwide. Despite the concern of salt content of KCJ, we observed the therapeutic benefits of KCJ in our adult subjects. These findings suggest that KCJ may have the potential to be developed as a whole food, nutraceutical or functional supplement for the management of obesity in some population.

## Conclusion

Decreased visceral adiposity and improved atherosclerosis indices were observed after overweight adults received KCJ supplementation for 12 weeks. The unique strength of this study was the observation of biological effect of KCJ, a whole food, in free-living overweight adults. However, the mechanisms responsible for these observed effects are yet to be elucidated.

## Competing interests

None of the above authors have conflicts of interest.

## Authors’ contributions

All authors had participated in this work with substantive contributions. Soo-Ran Kim Ji-Ae Yang, Hyang-Im Back, and Min-Gul Kim had the responsibility to carry out the daily responsibility of the RCT projects. Drs. Soo-Wan Chae, Won O Song, and Youn-Soo Cha had participated in biochemical analysis, interpretation of data. Dr. Su-Jin Jung carried out the statistical analysis. All authors read and approved the final manuscript.

## Funding sources

This study was supported by grants from the Ministry for Food, Agriculture, Forestry and Fisheries (20080410496-00) and research funds of Chonbuk National University in 2011.
